# Targeted therapies for renal cell carcinoma: more gains from using them again

**DOI:** 10.3747/co.v16i0.404

**Published:** 2009-05

**Authors:** H.K. Gan, B. Seruga, J.J. Knox

**Keywords:** Renal cell carcinoma, second-line therapy, sunitinib, sorafenib, everolimus, temsirolimus, bevacizumab

## Abstract

The development of molecularly targeted agents that inhibit pathways critical to the development of renal cell carcinoma has significantly improved outcomes in patients with these cancers. Compelling scientific and phase iii data have made the use of molecularly targeted agents the standard of care in first-line treatment. Now, available data show that re-treating patients with other tyrosine kinase inhibitors after they progress on sunitinib or sorafenib, or both, is beneficial. A large phase iii trial recently showed that, as compared with placebo, treatment with everolimus, an inhibitor of the mammalian target of rapamycin (mtor), almost halved the risk of progression (37% vs. 65%) and doubled the median progression-free survival (4 months vs. 2 months). Overall survival was not improved in that study, likely reflecting treatment crossover in the placebo arm, but these data position everolimus as the second-line standard of care. A consistent and growing body of literature also suggests that re-treatment with other kinase inhibitors that the patient has not previously encountered is a reasonable option. Outcomes of initial treatment with sunitinib or sorafenib (or both) should not deter the use of second-line targeted therapy, because the first-line use of targeted agents does not appear to be predictive of outcomes with second-line therapy. However, in view of poor absolute outcomes after second-line treatment and the benefits seen with rationally developed targeted agents in the first-line setting, enrolment of second- and subsequent-line patients in further trials would be preferable.

## 1. INTRODUCTION

**FIGURE 1:**
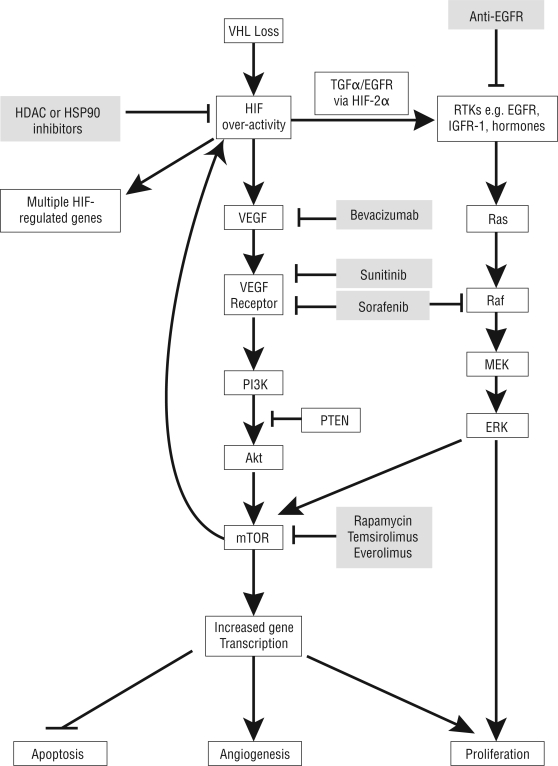
Simplified schema of signalling pathways that contribute to renal oncogenesis. vhl = Von Hippel–Lindau tumour-suppressor protein; egfr = epidermal growth factor receptor; hdac= histone deacetylase; Hsp90 = heat shock protein 90; hif = hypoxia-inducible factor; tgfα = transforming growth factor α; rtks = receptor tyrosine kinases; igfr-1 = insulin-like growth factor receptor 1; vegf = vascular endothelial growth factor; Raf = protein encoded by the murine leukemia viral oncogene homolog; Ras = ras signal transduction protein; pi3K = phosphoinositide-3-kinase; mek = mitogen-activated protein kinase; pten = protein encoded by the phosphatase and tensin homolog gene; erk = extracellular signal-regulated kinase; Akt = protein kinase B; mtor = mammalian target of rapamycin.

In 2005, renal cell carcinoma (rcc) was the ninth most common cancer in Canada, with an incidence of 1 per 10,000 population 1, and in that year, the disease was responsible for 2% of the total cost of cancer ($17 billion) 2. Until recently, patients with metastatic disease had limited treatment options, and their 5-year survival was approximately 20% 3. Moreover, tumours in rcc rarely responded to chemotherapy and radiotherapy 3. Cytokine therapy (interferon alfa or interleukin 2) had modest efficacy, with a response rate of 12% and a median survival of 13 months in a recent meta-analysis 4. However, since the development of molecularly targeted therapies (Table i) that inhibit the pathways important in the pathogenesis of rcc (Figure 1), the outlook has improved.

## 2. DISCUSSION

### 2.1 Epidemiology and Biology of rcc

Loss of the von Hippel–Lindau (vhl) tumour suppressor is a pivotal event in both hereditary and sporadic rcc (Figure 1) 3,11. The resultant overactivity of the hypoxia-inducible factor (hif) pathway leads to overexpression of a set of hypoxia-related genes [for example, vascular endothelial growth factor, platelet-derived growth factor B (pdgfrb)] and activation of a number of pathways [for example, the phosphoinositide-3-kinase (PI3K)/protein kinase B (Akt)/mammalian target of rapamycin (mtor) and *ras* signal transduction protein (Ras)/protein encoded by the murine leukemia viral oncogene homolog (Raf)/mitogen-activated protein kinase (Mek)/extracellular signal-regulated kinase (Erk) pathways] 12. Signalling through mtor is present in most clear-cell rcc ^13,14.^ In addition to effects on tumour proliferation, angiogenesis, and apoptosis, activation of mtor may also potentiate the activity of hif 12. Inhibition of mtor results in tumour inhibition *in vitro* 13 and *in vivo* 15.

Interestingly, the epithelial growth factor receptor (egfr) pathway is also important to renal oncogenesis. Expression of egfr occurs at high frequency in rcc (70%–90%) 16–18, and loss of vhl causes increased expression of transforming growth factor α 19 through hif-2α–dependent mechanisms 20–22. Inhibition of this dysregulated autocrine loop is sufficient to reduce or abolish tumour growth in multiple vhl–/– rcc cell lines 20,21.

### 2.2 First-line Treatment with a Targeted Agent

Treatment with a number of targeted agents (Table i) is now accepted as the standard of care in patients with metastatic rcc. In untreated patients, sunitinib 23, temsirolimus 24, and bevacizumab (when combined with interferon alfa) 25 were all found to be superior to interferon alfa alone. For example, Motzer *et al.* and Cella *et al.* 23,26 showed that patients treated with sunitinib experienced improved progression-free survival [pfs: 11 months vs. 5 months; hazard ratio (hr): 0.42; 95% confidence interval (ci): 0.32 to 0.54; *p* < 0.001), an improved objective response rate (orr: 31% vs. 6%; *p* < 0.001), and improved quality of life. Similar benefits were also seen in patients who had received prior cytokine therapy 27. In that group, treatment with sorafenib was superior to placebo with regard to pfs (6 months vs. 3 months; hr: 0.44; 95% ci: 0.35 to 0.55; *p* < 0.01) and the disease control rate (84% vs. 55%; *p* < 0.001). Quality of life was also improved in patients receiving sorafenib as compared with those receiving placebo 28. Only the egfr inhibitors failed to deliver substantial therapeutic benefit, either as single agents 17,18,29–31 or in combination with agents such as bevacizumab 32.

### 2.3 Second-line Treatment After Failure with a First-line Targeted Agent

Until recently, little evidence was available to guide therapy once patients had progressed on first-line treatment with a targeted agent. There is certainly a need to offer treatments to this patient population, not uncommonly encountered in clinical practice and often of appropriate performance status to tolerate more therapy. Re-treatment with another targeted agent has become commonplace practice despite the lack of prospective data 33, and a number of retrospective studies have now been published about this approach (Table ii). In all but two reports, the clinical benefit rate (complete response/partial response/stable disease) exceeded 50%, and in most cases, it reached 70% or better. In most reports, the median duration of benefit was 6 months or more. Re-treatment was generally well tolerated, with the most common grade 3 toxicities being fatigue, hypertension, and hand–foot syndrome.

Prior response to first-line targeted treatment did not predict response to second-line treatment 34–37. As with other targeted therapies 38, this lack of cross-resistance is thought to indicate incomplete suppression of tumour pathways with initial therapy. Further evidence of activity has been provided by the large prospective expanded-access programs of sorafenib 37 and sunitinib 39, which reported on 2488 and 2341 patients respectively. These programs showed that clinical benefit rates in these populations (84% and 52% respectively) were similar to those reported in phase iii trials. A substantial minority of patients previously exposed to targeted therapies also appeared to derive benefit from sunitinib and sorafenib in these programs.

The best evidence to date is provided by Motzer *et al*. 47, who reported a positive phase iii trial in this clinical setting. Patients who had progressed on sunitinib or sorafenib (or both) were randomized to either the mtor inhibitor everolimus (10 mg orally, once daily) or to placebo. Although patients from all prognostic groups were enrolled into the trial, approximately half the patients had an intermediate prognosis. The primary endpoint was pfs as determined by independent reviewers. The trial was halted early when an interim analysis indicated a substantial and significant difference in pfs in favour of the everolimus group. The progression rate was 37% in the everolimus group as compared with 65% in the placebo group (hr: 0.30; 95% ci: 0.22 to 0.40; *p* < 0.0001). Median pfs was 4.0 months (95% ci: 3.7 to 5.5 months) in the everolimus group as compared with 1.9 months (95% ci: 1.8 to 1.9 months) in the control group. Median overall survival (os) had not been reached in the everolimus arm (in excess of 10 months as compared with 8.8 months in the placebo group). The difference in os did not reach statistical significance, likely as a result of the planned crossover from placebo to everolimus on study. Although more stomatitis (40% vs. 8%), rash (25% vs. 4%), and fatigue (20% vs. 16%) occurred in the everolimus group, these side effects were mostly mild or moderate in severity. Pneumonitis (any grade) was detected in 22 patients in the everolimus group (8%), but only 8 patients 3 had grade 3 pneumonitis. Quality of life was equivalent in both study arms.

At this time, everolimus can reasonably be considered to be the preferred second-line treatment after initial failure of sunitinib or sorafenib. Although os is always preferable as the primary endpoint in phase iii trials, the use of pfs in the Motzer study is an acceptable surrogate, and the overall case supporting the efficacy of everolimus as a second-line treatment is strong 48. Although everolimus is not yet approved in Canada for metastatic rcc, temsirolimus could be considered in this setting, given that it is currently approved in Canada and has a similar mechanism of activity. Once completed, two phase iii trials currently in progress may affect this choice. The first randomizes patients to temsirolimus or sorafenib as second-line therapy after progression on sunitinib (search for “NCT00474786” at www.clinicaltrials.gov/ct2/search). The second randomizes patients who failed a previous systemic treatment, which may be a tyrosine kinase inhibitor, to either sorafenib or axitinib (search for “NCT00678392” at www.clinicaltrials.gov/ct2/search).

## 3. CONCLUSIONS

Inhibitors of multiple kinases such as sunitinib and sorafenib are now established as standard first-line therapy in patients with rcc. When disease progression occurs after such therapy, there is clearly more benefit to be gained by re-treating these patients with another targeted agent. Everolimus is the drug of choice at the present time, and we expect it to obtain approval for this indication in Canada soon. Until such time as approval is forthcoming, temsirolimus (if available) could be substituted, given its similar mechanism of action. It would also not be unreasonable to use any of the targeted agents in Table ii (if available) to treat patients with progressive rcc who have not previously been exposed to those agents. However, the benefits seen with rationally-developed targeted agents in the first-line setting strongly suggest that it is more appropriate to enrol those patients into clinical trials. Research priorities include the evaluation of predictive biomarkers to allow for patient enrichment, optimization of drug sequencing [concurrent vs. sequential, and simultaneous blockade at several points of the same pathway (vertical blockade) vs. blockade of several collateral pathways (horizontal blockade)], and identification of other effective drugs—for example, histone deacetylase 49,50.

## Figures and Tables

**TABLE I:** Selected targeted agents in the treatment of renal cell carcinoma (rcc)

*Name*	*Targets*	*Canadian regulatory status in rcc*
Tyrosine kinase inhibitors		
Sunitinib^5^ (Sutent^a^)	vegfr 1, 2, 3; pdgfra, b; Flt3; c-Kit; ret	Approved for patients with metastatic rcc of clear-cell histology and good or intermediate prognosis
Sorafenib 6,7 (Nexacar^b^)	Raf; vegfr2, 3; pdgfrb; Flt3; c-Kit; ret	Approved for patients with locally advanced or metastatic rcc who have failed, or are intolerant to, cytokine therapy
Temsirolimus 8 (Torisel^c^)	mtor	Approved for patients with metastatic rcc (not funded in all provinces)
Axitinib^9^ (Pfizer^a^)	vegfr1, 2, 3; pdgfra, b; c-Kit	Not approved
Everolimus 8 (Certican^d^)	mtor	Under consideration
Monoclonal antibodies		
Bevacizumab 10 (Avastin^e^)	vegf	Not approved

^a^ Pfizer Canada, Kirkland, QC.

^b^ Bayer HealthCare AG, Leverkusen, Germany.

^c^ Wyeth, Madison, NJ, U.S.A.

^d^ Novartis Pharmaceuticals, St. Louis, MO, U.S.A.

^e^ Genentech, San Francisco, CA, U.S.A., and Hoffmann–La Roche Ltd., Mississauga, ON.

vegfr = vascular endothelial growth factor receptor; pdgfra, b = platelet-derived growth factor receptor α and B; Flt3 = fms-related tyrosine kinase 3; Raf = protein encoded by the murine leukemia viral oncogene homolog; mtor = mammalian target of rapamycin; vegf = vascular endothelial growth factor.

**TABLE II:** Retrospective data regarding the efficacy of second-line targeted therapy

*Reference*	*Pts (*n*)*	*Targeted treatment*		*Clinical benefit*		*Benefit duration (months)*	*Frequency of grades 3 and 4 toxicities*
		*First-line*	*Second-line+*	*(%)*	*(CR/PR/SD) (*n*)*		
First-line sunitinib							
Sablin *et al.*, 2007 40	22	Sunitinib	Sorafenib	70	0/15/55	pfs: 6	na
Sepulveda, *et al.*, 2008 41	20	Sunitinib plus cytokines (100%)	Sorafenib	70	0/10/60	ttp: 7 os: 9	Fatigue (17%) Mucositis (9%) Hypertension (9%) hfs (9%)
Dudek *et al.*, 2009 42	20	Sunitinib plus cytokines (80%)	Sorafenib	35		ttp: 2	na
First-line sorafenib							
Sablin *et al.*, 2007 40	68	Sorafenib	Sunitinib	73	0/15/58	pfs: 7	na
Eichelberg *et al.*, 2008 43	30	Sorafenib plus cytokines (67%)	Sunitinib	55	0/11/44	pfs: 10	hfs (3%) Leucopenia (3%) Platelets (3%)
Dudek *et al.*, 2009 42	29	Sorafenib plus cytokines (55%)	Sunitinib	59	0/21/38	ttp: 5	na
First-line sorafenib + sunitinib							
Rini *et al.*, 2007 44	62	Sorafenib (100%) plus sunitinib (23%) plus cytokines (60%)	Axitinib	55	0/21/34		Fatigue (18%) Hypertension (16%) hfs (11%)
Dutcher *et al.*, 2008 45	59	Sorafenib plus sunitinib (24%) plus cytokines (64%)	Axitinib	100	0/32/68	pfs: approx. 8	Fatigue (13%) Hypertension (11%) hfs (11%)
Whorf *et al.*, 2008 46	29	Sorafenib (*n*=12), sunitinib (*n*=15), both (*n*=2)	Bevacizumab and everolimus	84	0/19/65	pfs: 11	Proteinuria (17%) Fatigue (7%) Stomatitis (7%)
First-line bevacizumab							
Knox *et al.*, 2007 37	197	Bevacizumab plus others (100%)	Sorafenib	81	0/3/78	na	na
Rini *et al.*, 2008^35^	61	Bevacizumab plus others (66%)	Sunitinib	82	0/23/59	pfs: 8 os: 12	Fatigue (34%) Hypertension (18%) hfs (10%)
Other first-line							
Rini *et al.*, 2008 36	37	Bevacizumab (*n*=15) or sunitinib (*n*=22) plus other anti-angiogenics (13%) plus cytokines or others (72%)	Sorafenib	43	0/3/40	pfs: 4	hfs (31%) Fatigue (17%) Hypertension (14%)
Tamaskar *et al.*, 2008 34	30	Miscellaneous (thalidomide, lenalidomide, bevacizumab, volociximab, AG13736, sorafenib, or sunitinib)	Sunitinib (*n*=16)	81	0/56/25	ttp: 10	na
			Sorafenib (*n*=14)	71	0/7/64		

Pts = patients; cr = complete response; pr = partial response; sd = stable disease; pfs = progression-free survival; na = not available; ttp = time to progression; os = overall survival; hfs = hand–foot syndrome.
